# Detection of Access Point Spoofing in the Wi-Fi Fingerprinting Based Positioning

**DOI:** 10.3390/s24237624

**Published:** 2024-11-28

**Authors:** Juraj Machaj, Clément Safon, Slavomír Matúška, Peter Brída

**Affiliations:** 1 Department of Multimedia and Information-Communication Technology, Faculty of Electrical Engineering and Information Technology, University of Zilina, Univerzitna 1, 01026 Zilina, Slovakia; slavomir.matuska@uniza.sk (S.M.); peter.brida@uniza.sk (P.B.); 2 Télécom SudParis, 9 rue Charles Fourier, 91011 Evry Cedex, France; clement.safon@telecom-sudparis.eu

**Keywords:** localization, positioning, spoofing, security, Wi-Fi fingerprinting

## Abstract

Indoor positioning based on Wi-Fi signals has gained a lot of attention lately. There are many advantages related to the use of Wi-Fi signals for positioning, including the availability of Wi-Fi access points in indoor environments and the integration of Wi-Fi transceivers into consumer devices. However, since Wi-Fi uses an unlicensed spectrum, anyone can create their own access points. Therefore, it is possible to affect the function of the localization system by spoofing signals from access points and thus alter positioning accuracy. Previously published works focused mainly on the evaluation of spoofing on localization systems and the detection of anomalies when updating the radio map. Spoofing mitigation solutions were proposed; however, their application to systems that use off-the-shelf items is not straightforward. In this paper filtering algorithms are proposed to minimize the impact of access point spoofing. The filtering was applied with a combination of the widely used K-Nearest Neighbours (KNN) localization algorithm and their performance is evaluated using the UJIIndoorLoc dataset. During the evaluation, the spoofing of Access Points was performed in two different scenarios and the number of spoofed access points ranged from 1 to 10. Based on the achieved results proposed SFKNN provided good detection of the spoofing and helped to reduce the mean localization error by 2–5 m, especially when the number of spoofed access points was higher.

## 1. Introduction

Indoor localization systems have attracted attention lately since these systems are crucial for the development of novel Location-Based Services (LBS) [1]. The implementation of LBS in health care, industry as well as other areas has the potential to improve user satisfaction and open new opportunities for service providers [2].

In recent years a vast number of localization systems based on data from various sensors have been proposed. The implementation of MEMS accelerometers and gyroscopes has allowed the development of Pedestrian Dead Reckoning (PDR) algorithms that could be executed on widely available consumer devices [3,4]. However, use in real-world scenarios is limited since the accuracy of the position estimates tends to decrease over time. Moreover, PDR algorithms require an estimate of the initial position and heading.

Another group of researchers focused on localization solutions based on the processing of audible and/or inaudible sound signals [5,6]. However, these systems may be limited by the parameters of mobile devices, as well as background noise that may vary significantly over time.

A lot of research effort was focused on image-based positioning [7,8]. However, these solutions usually require the installation of cameras in the area, which may lead to security concerns. Moreover, the use of camera systems is to some extent limited by regulations such as GDPR [9].

It could be concluded that the most effort was devoted to the development of localization solutions based on wireless networks [10,11]. Nowadays signals from radio networks are ubiquitous, and cellular networks as well as Wi-Fi have become crucial parts of our daily lives. Therefore, it makes sense to use these signals and consumer devices to develop a localization system.

Wi-Fi signals have become extremely popular for the development of localization solutions [12]. This is probably due to the fact that Wi-Fi works in ISM bands so that anyone can deploy their network. Moreover, most consumer devices, e.g., smartphones, tablets, or laptops, have Wi-Fi transceivers. Thus, using Wi-Fi signals would make the system cost-effective, especially from the consumer’s point of view.

However, the Wi-Fi standard was primarily developed for communication purposes. Therefore, most consumer devices are not able to measure signal propagation time, or angle of arrival accurately. Moreover, the majority of consumer devices do not support access to Channel State Information (CSI) measurements out-of-the-box. Thus, the most widely available option for positioning is to use Received Signal Strength (RSS) measurements, which are noisy due to fadings that are caused by obstacles as well as multi-path propagation. Consequently, traditional distance-based positioning does not work well with Wi-Fi signals [13].

The signal affected by fading, however, seems to be beneficial for fingerprinting algorithms [14]. These algorithms utilize a radio map, which describes the coverage of the area with signals from different Access Points (APs). This radio map is used to estimate the position of a device based on RSS samples collected by the mobile device. Fingerprinting localization can be implemented by a variety of algorithms, including deterministic and statistical algorithms as well as machine learning (ML) based estimators.

The use of Wi-Fi signals thus has great potential in indoor environments. However, a possible issue related to the real-world implementation of the fingerprinting-based localization system is represented by relatively easy spoofing of APs, which may lead to increased positioning errors [15]. This could represent a security issue, as users of the localization system may not find the right path to the desired destination [16]. Moreover, such false information about the position may lead users to some prohibited areas, for example, when the localization system is implemented in hospitals, airports, etc.

There have been studies focused on the detection of spoofed Wi-Fi signals, aimed at improving the resilience of the localization systems. The solutions found in the related work could be divided into two main groups.

The first group is based on cooperative measurements or gathering of signals from pre-installed infrastructure [15,17]. These measurements can help to detect attacks aimed at altering data in localization requests and have the potential to detect changes in visible APs. However, additional infrastructure is required, which results in increased cost of the system, especially when a large area is covered.

The second group of spoofing detection algorithms relies on the evaluation of signal properties. However, to make these algorithms work, it is required to gather CSI data [18,19], or a relatively large number of RSS samples at the same position [20]. Taking multiple RSS samples is not an issue; however, gathering 100 samples at one position introduces a significant delay in the position estimation process. Moreover, in contrast to RSS measurements, CSI measurements are not widely available on consumer devices. Thus, these approaches are not ideal for the implementation of a positioning system.

In this paper, approaches to reduce the impact of AP spoofing on fingerprinting localization will be proposed and tested. An evaluation of the proposed solutions will be performed on the UJIIndoorLoc dataset [21], with two different implementations of spoofing attacks. The main contributions of the paper can be summarized as follows:

- Three approaches to filter localization requests that include samples from spoofed APs were proposed.
- The filtering algorithms were applied to a well-known K-Nearest Neighbours (KNN) localization algorithm in order to evaluate their performance and impact on localization.
- The modified versions of the modified KNN were tested on a publicly available dataset, which was modified to include spoofed APs to evaluate the impact of an AP spoofing attack.

The rest of the paper is organized as follows: in Section 2 related work in fingerprinting-based positioning with Wi-Fi signals is described together with related work on AP spoofing. Section 3 describes proposed solutions for the detection of AP spoofing. The methodology used to create the testing datasets is summarized in Section 4. Achieved results are presented and discussed in Section 5 and Section 6 concludes the paper.

## 2. Related Work

In this section related work in fingerprint-based localization, as well as a summary of works dealing with spoofing in wireless positioning systems based on Wi-Fi is presented. The description of fingerprinting algorithms is focused on the main principles as well as a description of the KNN algorithm, which was implemented in the testing scenarios as a baseline. The section includes an overview of ML-based positioning algorithms and a comparison of their performance with KNN on the UJIIndoorLoc dataset.

### 2.1. Fingerprinting Localization

The fingerprinting localization gained a lot of attention in indoor positioning due to the fact that it does not require the estimation of pseudo-ranges from signal parameters like RSS or Time of Arrival (ToA). These parameters are affected by the signal propagation in the dynamic environment, thus pseudo-ranges cannot provide accurate information about the distance between wireless devices with high reliability. Fingerprinting on the other hand can benefit from the signal attenuation caused by the obstacles in the indoor environment.

Fingerprinting localization algorithms can be divided into three major groups, deterministic algorithms, statistical algorithms and neural network-based [12,13]. Both deterministic and statistical algorithms use data in the radio map database to estimate the position of the mobile device based on the parameters provided in the localization requests. On the other hand, solutions based on neural networks create a model based on the calibration data, i.e., the radio map, and use this model to estimate the position of the mobile device.

The general principle of fingerprinting localization can be divided into two stages. The first stage is the calibration of the system. During this stage, the calibration measurements are performed in the area of interest [22]. The area is divided into regions represented by reference points. At each reference point signal parameters, typically RSS samples, are collected. These represent fingerprints that are stored in the radio map database. Recent works also focus on the use of CSI data for positioning [23]. The advantage of CSI over RSS lies in more detailed information about the environment, thus CSI has the potential to improve localization accuracy. On the other hand, current devices do not support access to CSI measurements out of the box; therefore, some modification of devices is required to support CSI measurements. The calibration phase also includes the pre-processing of data, the creation of a radio map database for deterministic or statistical algorithms, and the training of models for neural network-based implementations.

The second stage represents the operation of the localization system. During this stage, the system is used to estimate the positions of mobile devices. The mobile device with an unknown position can measure signal parameters; in this case, the RSS samples from surrounding APs, and send them to the localization server. The localization server utilizes an implemented localization algorithm to estimate the position of the mobile device.

This paper deals with the implementation of a deterministic KNN algorithm, thus the result of the calibration phase is assumed to be a radio map. The KNN algorithm used in the experiments is based on an assumption that RSS samples collected by the mobile device are not random but depend on the position of the mobile device [13]. Thus, the position of the mobile device $\tilde{x}$ can be estimated as follows:

(1) $$\tilde{x}=\frac{\sum_{n=1}^{M}\omega_{n}\times \gamma_{n}}{\sum_{n=1}^{M}\omega_{n}},$$

where *M* is the number of reference points in the radio map, $\gamma_{n}$ represents the position of *n*-th reference point and $\omega_{n}$ stands for the weight assigned to the *n*-th reference point. The weights $\omega_{n}$ are estimated as the inverse value of Euclidean distance between RSS samples received from the mobile device and RSS samples stored in the radio map database.

In the case of the KNN algorithm, the *k* highest weights are set to 1 and the rest of the weights are set to 0. Thus, the position estimate is given as an average value of *k* reference points with the highest similarity of fingerprints compared to the fingerprint received from the mobile device [10].

Song et al. introduced the localization system CNNLoc [24]; in the system, the position is estimated using a one-dimensional Convolutional Neural Network (CNN) from Wi-Fi fingerprints. They proposed the use of a Stacked Auto-Encoder (SAE) to extract key features of RSS samples in a radio map database.

An indoor localization framework focused on the compression-friendly implementation of a convolutional autoencoder with a CNN called CHISEL was proposed by Wang et al. [25]. The authors proposed a localization solution that allows compression and reduces the footprint of the model, making it more suitable for embedded devices. Based on the achieved results, the model can be more than five times smaller and achieve higher accuracy than classical ML models.

The Wi-Fi-based localization system SE-Loc was proposed by Ye et al. [26]. The proposed system uses a semi-supervised learning-based technique to enhance the performance of the localization system. The authors divided the architecture of the localization system into two parts. The first part is responsible for the selection of APs based on correlation and generation of fingerprint-image. The second part consists of a deep learning model, which uses a denoising autoencoder in combination with a CNN.

An extreme learning machine autoencoder was used to reduce the dimension of input data in the EA-CNN model proposed by Alitaleshi et al. [27]. The proposed method helps to bypass the computational complexity of conventional stacked autoecoders and increases the accuracy of position estimates. The authors also implemented a data augmentation strategy by adding noise to the model; this approach was used to increase the robustness of the positioning algorithm.

Pei et al. [28] proposed the FTLoc localization method, which utilizes the sequence-to-sequence network structure. The authors proposed to use a combination of the attention and the learnable filter. The proposed solution consists of two modules. The first module is a feature encoder, which includes a self-attention module and a learnable filter module is used to extract multilevel features from RSS samples. These multilevel features should reduce the impact of the heterogeneity of devices. On the other hand, the second module, named the location decoder, consists of two attention modules and is used to estimate the positions of mobile nodes.

A hybrid deep learning-based localization method was proposed by Turgut et al. [29]. The authors used a Long-Short-Term Memory (LSTM) to capture signal feature dependencies over long-term periods. Moreover, a CNN was used to extract local spatial signal patterns. Furthermore, particle filter and sparse autoencoder were implemented for filtering and dimension scaling on the RSS data.

Shen et al. proposed a positioning method called virtual feature maps and contrastive learning-enhanced indoor positioning (VF-CLIP) [30]. The method uses RSS samples and is aimed at reducing the repetitive site survey overhead. The VF-CLIP reconstructs fingerprints by incorporating newly added APs using a deep neural network fine-tuning technique.

Narasimman and Alphones proposed a positioning solution that utilizes a new dimensionality reduction technique as well as input and output normalization schemes for the transferability of the model in [31]. The solution uses common tree-based techniques, i.e., random forests and extreme gradient boosting, to estimate the position of the mobile device. According to the authors, the proposed solution can achieve similar results as state-of-the-art techniques without the need for optimization.

The use of the Graph Neural Network (GNN) for indoor positioning was proposed by Wang et al. in [32]. The proposed solution uses graph topology to establish neighbor relationships and solves misaligned features. Authors use GNN to improve the stability of potential neighbor features, which are used for adaptation to dynamic environmental changes.

It should be noted that there is a plethora of algorithms that can be used for position estimation using Wi-Fi [33,34,35]; however, the description of these algorithms is beyond the scope of this paper.

The comparison of the accuracy of the KNN algorithm and methods based on machine learning on the UJIIndoorLoc dataset is presented in Table 1. From the table, it can be seen, that the KNN algorithm, used as a baseline for implementation of proposed filters, can achieve accuracy similar to the CNN-based localization methods. It is clear that more advanced ML-based localization schemes can achieve higher accuracy; however, it should be noted that some of the results presented in the table take into account errors only in 2D space. Moreover, some of these methods use the augmentation of training data; with the implementation of data augmentation and/or interpolation methods on the training dataset, the performance of KNN could be improved as well.

However, the design of the abovementioned localization algorithms does not consider AP spoofing, thus it can be assumed that the majority of algorithms will be affected by spoofing in a similar way as the KNN algorithm since all algorithms perform classification tasks.

The proposed filtering approaches were tested on the KNN algorithm since this algorithm is deterministic, thus it should not be affected by the parameters of the dataset. In contrast, the ML-based approaches may be affected by overfitting. In such cases, the resulting model is significantly affected by the properties of the dataset, leading to overestimated accuracy and poor generalization.

### 2.2. Spoofing of Access Points

Tippenhauer et al. studied the security of public WLAN-based positioning systems in [36]. The experiments were performed on the Skyhook positioning system. The presented results showed that it was possible to perform location spoofing as well as location database manipulation attacks and concluded that more work should be focused on the development of secure positioning solutions.

Matte et al. in [37] extended the work of Tippenhauer et al. and presented an attack that could be used to obtain the identity of a Wi-Fi-based localization system user by physical proximity-based attack. The authors demonstrated that the privacy of the user may be at risk if position spoofing in wireless positioning systems is not prevented.

Furthermore, Wang et al. presented means to spoof position based on open source projects in [38]. The work focused not only on Wi-Fi-based positioning but GNSS spoofing was also considered. The authors managed to alter position estimates from the localization implemented in Apple smartphones.

The related work mentioned above has concluded that APs can be spoofed and provided some insights into how these spoofed APs affect positioning accuracy. However, countermeasures dealing with the spoofing were not implemented and evaluated.

### 2.3. Detection of Spoofed Access Points

Li et al. presented a study about the security issues in Wi-Fi-based indoor positioning systems that utilize crowdsourced data in [39]. Three different attacks were presented and evaluated. In the first attack the attacker generates fake RSS samples randomly, in the second attack the attacker adds some noise to the valid RSS samples and in the last attack the attacker changes the mapping between fingerprints and their locations. Moreover, spatial trustworthiness metrics for the generation of a radio map database from crowdsourced data were proposed.

It should be noted that this work deals with the detection of false data in the crowd-sourced radio map. Although some of the principles could be used during the positioning, it would affect the response time since iterative algorithms were implemented in the proposed solution. This is not an issue for the radio map creation process, since the radio map does not need to be constructed in real-time.

Luo et al. studied the instability of RSS and its impact on the performance of the fingerprinting localization system in [40]. The impact of impaired, broken and newly-introduced APs on the localization system was evaluated. The authors proposed a voting mechanism, which identifies factors that impact RSS values and extracts the trust factor of the fingerprint. The authors proved that the proposed algorithm can identify impaired APs, i.e., APs that do not work properly.

The algorithm for the detection of impaired APs was tested in a scenario where AP power decreased significantly or AP was turned off. However, using this algorithm in a more complex environment to detect spoofing of APs was not considered in the work.

Ye et al. [15] showed a privacy attack model based on the spoofing of location in mobile social network services. The authors also proposed a mechanism to defend against such attacks based on base station tags. They were used for the authentication of the spatial-temporal property of geolocation and prevented spoofing attacks on a WLAN-based positioning system.

Although the proposed solution worked well, it should be noted that the test was carried out at two locations that were in different cities; therefore, the use of base station tags for geolocation could make sense. However, if the attack is performed inside a smaller area, e.g., campus, hospital, airport, etc., the proposed solution may not provide a robust solution.

Restuccia et al proposed Location Validation System (LVS) in [41]. The proposed LVS should secure location-based services from location spoofing attacks. The proposed solution is based on validation rounds, in each round the position of the user is validated by the neighboring nodes and the reputation of nodes is estimated to detect nodes with spoofed positions. The proposed solution relies on the cooperation of neighboring nodes, which can be represented by smartphones with the capability to create Wi-Fi hotspots.

The use of the spatial correlation of RSS from nodes to detect the spoofing attacks was proposed by Yang et al. [17]. The proposed solution is based on the assumption that the RSS measured at a set of landmarks is closely related to the location of the transmitter and distance from the landmark. Thus, the spatial RSS-based correlation of RSS samples from landmarks with RSS samples in localization requests could be used to detect the spoofing of Wi-Fi signals. The main drawback of the scheme is that it requires a set of landmarks to collect RSS samples, thus increasing the cost of infrastructure. Moreover, it can detect when localization requests are altered by an attacker. However, signals from spoofed APs could be received by both localized devices and landmarks.

The improved user authentication scheme based on CSI measurements was proposed by Liu et al. in [18]. Although the scheme is primarily used for the authentication of Wi-Fi clients, it can be used also for spoofing detection. The proposed solution is based on the assumption that CSI measurements of each device have unique spatial characteristics. Moreover, CSI measurements have a strong spatial correlation with the position of the device. The main problem is that most off-the-shelf devices do not support access to the CSI measurements without software modification.

A system that uses deep learning to detect virtual MAC spoofing attacks in wireless networks was proposed by Jiang et al. [19]. The MAC spoofing attack is detected by a deep CNN, which utilizes CSI measurements to extract the physical features. These features can be used to detect spoofing attacks. The authors report an average detection accuracy of 95%.

Location spoofing attack detection based on the Wasserstein metric was introduced by Tian et al. [20]. The detection of spoofed APs is based on the assumption that wireless signals have different features when they are transmitted by different devices. Since multiple fake APs may be created using a single device, these signals will have similar features and thus can be detected. The proposed solution is based on RSS measurements; however, it requires a relatively large number of samples to estimate the features of the signals. Moreover, the samples should be collected at a static position, thus the solution would introduce a significant delay in the localization process.

As can be seen from the summary of related works shown in Table 2, the problem of spoofing in wireless networks is a topic that attracts a lot of attention. However, there is still a lack of solutions that could provide some protection in case of a spoofing attack without the need for additional data, i.e., signals collected by landmarks or CSI measurements. Previously proposed solutions for the detection of anomalies when updating radio maps use iterative algorithms, so their application in real-time position estimation is limited. On the other hand, the voting algorithm for impaired AP detection requires updates of the radio map, and voting relies on changes in RSS, thus it is not optimal for spoofing detection, where the signals may be stable. The solution based on geolocation from cellular networks has some potential, but may not work if spoofing is performed within the same geographical area, e.g., university campuses, hospitals, and airports.

In this work, filtering approaches are proposed to remove localization requests affected by the spoofing of Wi-Fi signals. The proposed solutions do not require any additional hardware and only utilize data, which are readily available in the training dataset, which was collected during the calibration phase of fingerprinting-based localization.

## 3. Proposed Filtering Algorithms for Protection Against Spoofing

In this paper, the basic idea for the protection against AP spoofing is to implement a filter for localization requests, which would detect anomalies in the localization requests received from the mobile device. In the spoofing attack, the attacker is transmitting signals from fake APs, these fake APs are using MAC addresses of APs that are stored in the radio map, but at different locations. In case the MAC address of a fake AP used by the attacker is not present in the radio map the KNN algorithm is not affected, since the RSS from such an AP would not affect the Euclidean distance used for position estimation.

To detect the anomalies in the positioning request, three different filtering approaches were proposed. The first approach was to select the data from the radio map only if there is some overlap between the APs detected at the given reference point and the APs detected in the request. We refer to this approach as Shared APs, since we are searching for an RP with the same APs as in the localization request. The implementation with the KNN algorithm is referred to as KNNSAP (KNN with Shared APs).

The second proposed approach is to filter the data in the radio map based on the difference between data stored in the radio map database and in the request from the mobile device, referred to as Unshared APs. Similarly, the modified KNN algorithm will be referred to as KNNUAP.

Finally, the third approach takes into account the estimated geographical position of APs. These estimates can be calculated from the data stored in the radio map. This information will be used to detect the anomalies in the RSS samples included in the localization requests. This approach is referred to as Spatial Filter and the implementation with KNN will be referred to as SFKNN.

It should be noted that proposed filtering approaches, i.e., SAP, UAP and SF can be implemented with any other localization algorithm during the pre-processing of localization requests at the localization server. The first two approaches, i.e., SAP and UAP, can be used to extract only part of reference points from the database and thus reduce the computational complexity of the localization algorithm since localization is performed with a subset of the database. Moreover, these algorithms will discard localization requests that do not have the required similarity (SAP) or have high differences (UAP) when compared to data in the training dataset. The third filtering approach can be used to discard localization requests that include APs with non-overlapping coverage areas. The coverage areas can be estimated using the data from the training dataset.

### 3.1. KNN with Shared Access Points

The KNN with Shared Access Points (KNNSAP) localization algorithm is based on the assumption that the position of a mobile device should only be estimated if there is a certain overlap between APs detected in the localization request and APs fingerprint from the database. The pseudocode for the KNNSAP is presented in Algorithm 1.

This algorithm is implemented in a way that Euclidean distance is only computed with a subset of fingerprints stored in the radio map. The subset of the fingerprints is defined by the minimum required overlap of the detected APs.

By implementing this requirement, it is also possible to reduce the complexity of the localization process, since the number of calculations of Euclidean distance is reduced. The calculation of distance is a more complex process than the selection of data from the database.
**Algorithm 1** KNNSAP  1:Inputs: (LocalizationRequest, RadioMapDatabase, MinOverlapThreshold)  2:Extract valid APs from LocalizationRequest to DetectedAPs  3:ValidFingerprints = []  4:**for** fingerprint in RadioMapDatabase **do**  5:    Calculate the overlap between detected APs and APs in fingerprint  6:    **if** overlap ≥ MinOverlapThreshold **then**  7:        ValidFingerprints.append(fingerprint)  8:    **end if**  9:**end for**10:**if** length(ValidFingerprints) < 1 **then**11:    Return: Localization failed12:**else**13:    EstimatedPosition = KNN(LocalizationRequest, ValidFingerprints)14:    Use the KNN algorithm to estimate position based on valid fingerprints15:    Euclidean distance is computed only with valid fingerprints16:    Return EstimatedPosition17:**end if**

It should be noted here that the implementation of KNNSAP may result in a higher number of failed localizations in cases when the threshold for the minimum overlap is set to a higher value. The threshold should be set up based on the minimum number of APs detected in the radio map. Moreover, in real-world conditions, some APs that are present in the radio map may be unreachable due to dynamic radio channel conditions and signal blockage.

The fingerprints extracted from the radio map database are further processed by the KNN algorithm, in order to estimate the position of the mobile device.

### 3.2. KNN with Unshared Access Points

The KNN with Unshared Access Points (KNNAUP) algorithm is based on a similar principle as KNNSAP, as can be seen from the pseudocode presented in Algorithm 2.
**Algorithm 2** KNNUAP  1:Inputs: (LocalizationRequest, RadioMapDatabase, MaxDifferenceThreshold)  2:Extract valid APs from LocalizationRequest to DetectedAPs  3:ValidFingerprints = []  4:**for** fingerprint in RadioMapDatabase **do**  5:    Calculate the difference between detected APs and APs in fingerprint  6:    **if** difference ≤ MaxDifferenceThreshold **then**  7:        ValidFingerprints.append(fingerprint)  8:    **end if**  9:**end for**10:**if** length(ValidFingerprints) < 1 **then**11:    Return: Localization failed12:**else**13:    EstimatedPosition = KNN(LocalizationRequest, ValidFingerprints)14:    Use the KNN algorithm to estimate position based on valid fingerprints15:    Euclidean distance is computed only with valid fingerprints16:    Return EstimatedPosition17:**end if**

The main difference is that, when selecting fingerprints from the radio map database the constraint is given by the maximum difference between the APs detected by the mobile device and APs stored in the database.

This constraint should help remove fingerprints that include data from an area that is different from the position of the mobile device. Similarly to KNNSAP, KNNUAP will also reduce complexity since only part of the radio map will be used for the calculation of Euclidean distance.

In this case, the performance of the algorithm will be affected by the threshold that defines the maximum allowed difference between APs in localization requests and APs in the radio map database. It is important to note here that if the threshold is too small it will affect the success rate of the position estimation process.

This is due to the fact that some APs may be detected with low RSS values even in areas where these were not detected during calibration measurements, and therefore, are not stored in the radio map. This can be caused by the use of different devices with different antenna gains and different sensitivity, as well as by changes in radio channel parameters, shadowing and multipath propagation.

After the selection of fingerprints from the radio map, the position is estimated with the KNN algorithm.

### 3.3. Spatially Filtered KNN

The proposed SFKNN algorithm for spoofing detection was developed to detect beacon signals from the fake APs. The basic idea behind the algorithm is to detect beacons transmitted by APs that are not supposed to be in the user’s vicinity. If such APs can be identified, the spoofing attack will not be successful. Otherwise, the position estimate could be less precise or even force the user to receive a position desired by the attacker.

The primary approach to counter these attacks is to implement a filter to detect fingerprints corrupted by AP spoofing and exclude them. The filter implemented in SFKNN is based on the assumption that each access point covers just a limited area. Thus, certain combinations of detected APs could be considered invalid since detected APs are too distant according to the data stored in the radio map. The pseudocode of SFKNN implementation is presented in Algorithm 3.
**Algorithm 3** SFKNN  1:Inputs: (LocalizationRequest, RadioMapDatabase)  2:Load metadata from RadioMapDatabase including estimated AP.position and AP.range of all APs  3:Extract valid APs from LocalizationRequest to DetectedAPs  4:CheckForSpoofing(DetectedAPs, metadata)  5:**for** each combination of APs in DetectedAPs **do**  6:    **if** distance(AP1.position, AP2.position) > (AP1.range + AP2.range) **then**  7:        Return: Spoofing detected  8:    **end if**  9:**end for**10:**if** Spoofing detected **then**11:    Return: Localization failed - Spoofing detected12:**else**13:    EstimatedPosition = KNN(LocalizationRequest, RadioMapDatabase)14:    Use the KNN algorithm to estimate position based on valid fingerprints15:    Euclidean distance is computed only with valid fingerprints16:    Return: EstimatedPosition17:**end if**

It is assumed that there should be an overlap between the areas covered by the APs detected by the mobile device during the positioning stage; otherwise, the spoofing attack is detected.

In order to implement this solution it is required to generate metadata from the radio map, or training dataset. It is assumed that the position of APs in the area is not available. The position of APs in the metadata is estimated as the average weighted position according to the RSS data and positions of reference points stored in the radio map dataset. Furthermore, the maximum coverage range can be estimated from the dataset and the estimated position of the AP.

**Figure 1:**
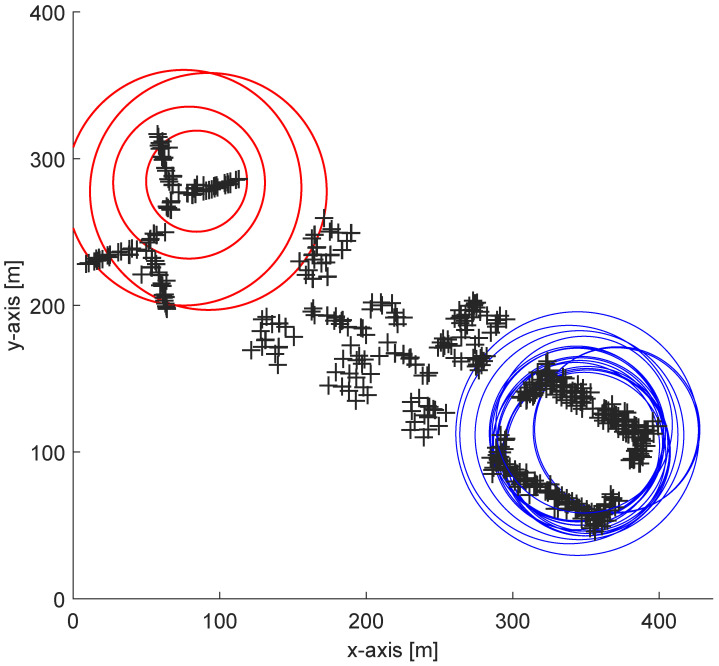
Visualization of spoofing detection principle implemented in SFKNN on localization request with spoofed APs.

Based on these metadata, it is possible to recognize problems in detected APs. To visualize the principle of the proposed solution it is possible to create a map with circles representing the range of each detected AP as can be seen in Figure 1.

In the figure, the blue circles represent the majority of APs that cover the common area and were detected during the positioning stage. On the other hand, red circles represent APs that do not overlap with the majority of detected APs. Therefore, a localization request that includes all these APs is assumed to include spoofed signals.

If the fingerprint is considered to be valid, i.e., no spoofing was detected by the algorithm, the KNN localization algorithm is used to estimate the position. We did not consider modified KNNSAP and KNNUAP at this stage. Therefore, the whole radio map was used to calculate Euclidean distances and position estimation.

## 4. Testing Scenarios

The evaluation of the algorithms was performed with the UJIIndoorLoc dataset. The dataset covers a surface of 108,703 m^2^ including three buildings with four to five floors. The measurements for the radio map data were collected at 933 different positions with approximately 21 samples per reference point. The total number of measurements, i.e., the number of fingerprints at the reference points in the radio map database was 19,938. On the other hand, for testing purposes, 1111 requests were used to estimate position in the dataset. The testing dataset was collected 4 months after the creation of the radio map. The total number of APs detected in the dataset was 520 and the data were collected by multiple users and 25 different models of mobile devices [21].

Although the dataset includes 520 APs, it is important to note that not all APs are detected at all fingerprints, i.e., RSS samples collected at reference points and testing points. The median number of detected APs in the training data, i.e., radio map database, is 17, while the minimum of detected APs is 1 and the maximum is 51. For testing data, the median number of detected APs is 15, with a minimum of 1 and a maximum of 35. In the testing dataset, 17% of localization requests include samples from 10 or fewer APs, while 74% include 20 or fewer APs. Therefore, it can be assumed that spoofing of 10 or fewer APs should have a significant impact on the performance of the localization system.

It should be noted that the results of the localization algorithms are affected by these parameters of the dataset. However, the dataset was created for the indoor localization competition at the IPIN 2015 conference and is still widely used for evaluation of localization algorithms [31,32,42], since it is one of few datasets that cover multiple multi-story buildings. In this work we do not focus on the development of novel positioning algorithms; therefore, we have only investigated the impact of filtering in comparison to the performance of the unmodified KNN algorithm. However, the proposed filtering solutions can be easily implemented to filter localization requests for other localization algorithms, since these only require access to the training dataset.

The AP spoofing attack on a fingerprinting-based positioning system is a problem that can introduce increased localization errors and lead users to believe they are at a different location, especially in complex indoor environments. To achieve this, attackers send fake AP beacons that affect the RSS fingerprints sent by the user’s device to the server.

In order to analyze the performance of the proposed algorithms alongside the original testing dataset, datasets with added RSS values that represent spoofing were used in the evaluation. Two different datasets were created to simulate AP spoofing scenarios. In both scenarios, datasets were created with different numbers of spoofed APs ranging from 1 to 10 for each positioning request, since in real-world conditions the attacker can send beacons from multiple fake APs.

In Scenario 1, we assume that the attacker knows some of the MAC addresses of APs stored in the radio map. The attack is performed by sending beacons with those MAC addresses. This attack represents the simplest way of implementation, as we assume the attacker will just plant some random APs in the area without setting their powers to emulate realistic fingerprints from the radio map database.

It should be noted here that users, who could represent attackers, do not have direct access to the data in the radio map database. In this scenario, a given number of fake APs was randomly selected and these were given a random RSS value between −70 dBm and −30 dBm. Therefore, the attacker does not necessarily have information about the parameters of APs required for a more sophisticated attack.

On the other hand, Scenario 2 represents a more severe situation, where the number of spoofed APs increases. In this case, we assume that the attacker has some knowledge about the RSS values that would represent a valid localization request from an area far from the real position. The attacker is trying to recreate measurements he obtained in another place and thus affects the operation of the localization system.

In this scenario, in order to create the dataset with spoofing attacks a fingerprint from another building was selected and RSS values from a number of APs were copied to the original localization request. By randomly selecting data from a different building, we can assume that the spoofed APs will be different than the APs normally detected by the mobile device.

In this case, the attacker sends beacons with MAC addresses with specific RSS values, and the user device will receive a (partial) copy of the fingerprint from another location, i.e., another building. The attacker aims to make the user believe they are in a specific place, where the fake APs are normally located. Due to the nature of the used dataset, in some situations, the user may receive more beacons from fake APs than from real APs. Thus, it can be assumed that the impact on the localization performance will be significant.

In the performed tests, the number of reference points *k* used for the estimation of the position was set to 4. This number of reference points usually provides good results for both KNN and WKNN algorithms [13]. All the parameters for both experimental scenarios are summarized in Table 3.

Based on preliminary experiments, the limit for the minimum overlap between APs with valid RSS samples in a positioning request and radio map database, which is used to filter out radio maps for KNNSAP, was set to 5. It should be noted that a higher limit could improve the localization accuracy and performance of the algorithm. However, it would reduce the number of successful localizations in cases when the number of detected APs is low.

On the other hand, the limit for the maximum difference between APs with valid RSS samples in a positioning request and radio map database used to filter out radio maps for KNNUAP was set to 15. Here, it should be noted that low values could result in problems even under normal conditions, since sometimes signals from distant APs can be detected with low RSS values and thus the number of detected APs can increase dramatically. Moreover, taking into account that the time difference between the training and testing database is 4 months, some new APs could be detected in the area.

## 5. Achieved Results and Discussion

### 5.1. Achieved Results

To analyze the localization performance of all proposed approaches for the detection of spoofing, the boxplot charts will be used to analyze localization errors. The boxplot provides a visual representation of the dataset static, the median value is shown by the line inside the box. The edges of the box itself mark values for lower and upper quartiles, i.e., 25th and 75th percentiles of the achieved localization errors. Moreover, the lowest and the highest non-outlier values are marked by the whiskers, represented by the black lines that extend above and below each box. The boxplots also include notches represented by shaded regions around the median value (50th percentile), with 5% significance levels, thus helping with the comparison of results.

The localization error is given as the Euclidean distance between the position provided in the testing dataset and the position estimated by the localization algorithm. Since the dataset provides height information as an integer value defining floor in a building, in the estimation of the error we assume a floor height of 3 m as a penalty for incorrect floor detection. The localization error is given by the following:

(2) $$E=\sqrt{{\left(x_{gt}-x_{est}\right)}^{2}+{\left(y_{gt}-y_{est}\right)}^{2}}+3\cdot \left(z_{gt}-z_{est}\right),$$

where $\left[x_{gt},y_{gt},z_{gt}\right]$ represents coordinates of ground truth position and $\left[x_{est},y_{est},z_{est}\right]$ stands for coordinates estimated by the localization algorithm.

**Figure 2:**
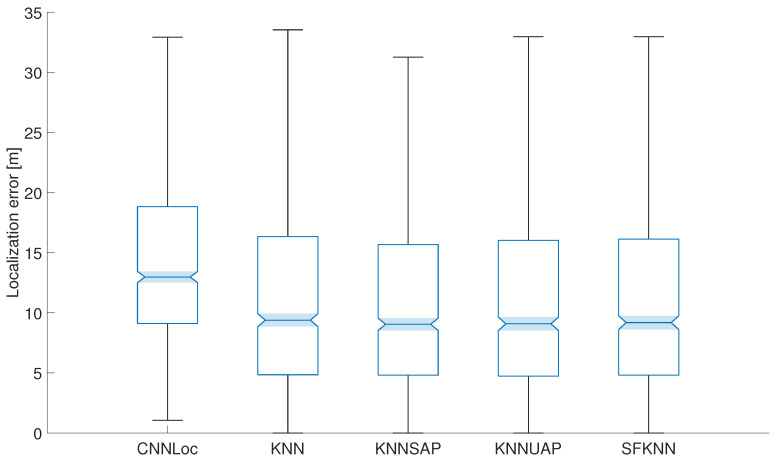
Localization errors achieved by proposed solutions without spoofing of APs.

In the first step, we decided to evaluate the impact of the proposed solutions on localization accuracy without AP spoofing, i.e., with the original testing dataset from the UJIIndoorloc dataset. The results achieved for all the algorithms are presented in Figure 2.

From the achieved results it can be seen that the localization errors achieved by all modifications of the KNN algorithm are very close to each other. On the other hand, CNNLoc achieved slightly worse results in the scenario without spoofing. This was expected since the average error achieved by CNNLoc and shown in Table 1 was slightly higher compared to the KNN algorithm. The results achieved by the KNNSAP reached a slightly lower maximum error, which may be given by the fact that the algorithm estimated the position by using only the reference points that have an overlap of a minimum of five APs; therefore, it was possible to reduce the positioning error in some cases.

**Figure 3:**
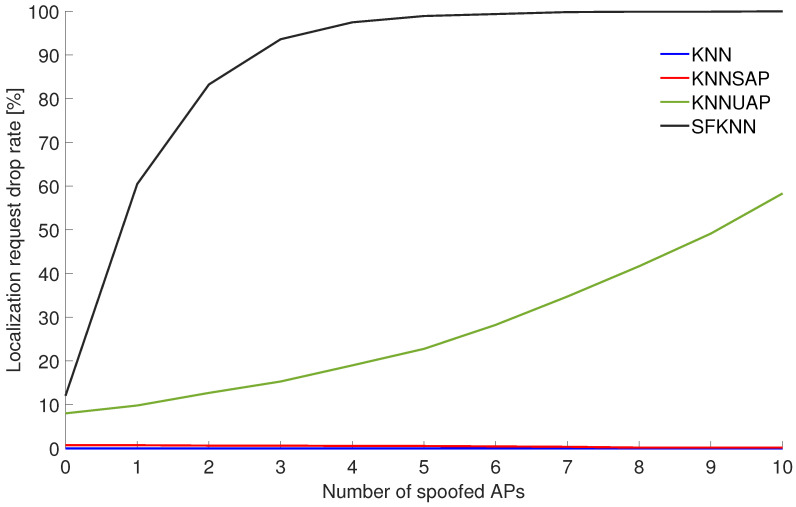
Localization request drop rate for different numbers of spoofed APs in Scenario 1.

The next step was the evaluation of the performance of proposed algorithms in the scenarios with the spoofing of APs. Figure 3 shows the localization request drop rate, i.e., denial of positioning, because conditions were not met for the given algorithm.

From the figure, it can be seen that around 10% of the requests were denied by the KNNAUP and SFKNN. This represents false positive detection of spoofing. However, it is important to note that part of these localization requests include RSS measurements from a low number of APs.

It can also be seen that in Scenario 1 the SFKNN declines more than 90% of localization requests when three or more APs are spoofed. On the other hand, the basic KNN algorithm as well as the KNNSAP algorithm do not drop any localization requests.

In order to see the impact of the spoofed APs on localization accuracy, the mean localization error achieved by all the algorithms for a number of spoofed APs ranging between 1 and 10 is summarized in Table 4.

From the table, it can be seen that the impact of the spoofing of APs is relatively small. Moreover, it can be concluded that KNNSAP achieved the lowest positioning error, except when the number of spoofed APs was 6. In such a case, SFKNN outperformed the KNNSAP. However, it is important to note that in this case, the mean error is calculated only from two successful position estimates. Moreover, it can be seen that CNNLoc achieved much lower accuracy when AP spoofing was introduced. Interestingly, the KNN algorithm seems to be relatively stable compared to the CNNLoc method.

**Figure 4:**
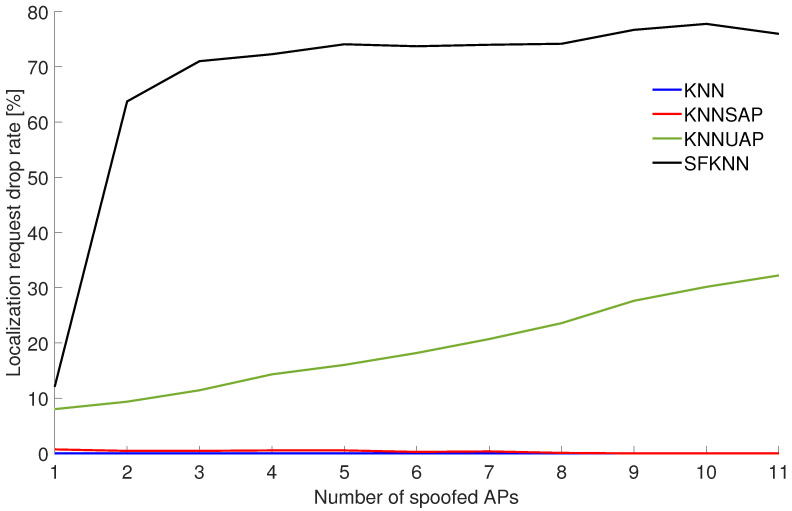
Localization request drop rate for different numbers of spoofed APs in Scenario 2.

Similarly, the same experiment was performed in Scenario 2, where spoofed APs have real-world values from different locations, represented by different buildings. Figure 4 depicts the localization request drop rate for Scenario 2.

In this case, it can be seen that similarly to Scenario 1, KNN and KNNSAP are not dropping localization requests when the number of spoofed APs increases. Moreover, the SFKNN algorithm again achieved the highest number of dropped requests. However, it should be noted that in Scenario 2 the the drop rate did not achieve 100%, which is due to the fact that some requests included spoofed APs from a nearby building and thus the algorithm was not able to detect the spoofing.

Mean localization errors achieved in Scenario 2 are summarized in Table 5. From the table, it can be seen that this scenario had a higher impact on the mean localization error of the implemented algorithms since realistic fingerprints were used for spoofing.

KNNSAP achieved the lowest mean localization error when the number of spoofed APs was low. When the number of spoofed APs increased the localization error was similar to basic KNN.

On the other hand, SFKNN achieved the second-lowest mean localization error when the number of spoofed APs was low. Moreover, the impact of the higher number of spoofed APs was the smallest for the SFKNN algorithm. Thus, with the highest number of spoofed APs, the SFKNN algorithm achieved the lowest localization errors.

Similar to Scenario 1, the CNNLoc achieved much worse localization performance when spoofed APs were introduced. Therefore, it can be concluded that the spoofing of APs has a significant impact on ML-based methods. Thus, the implementation of filtering to reduce the impact of spoofing should be considered.

**Figure 5:**
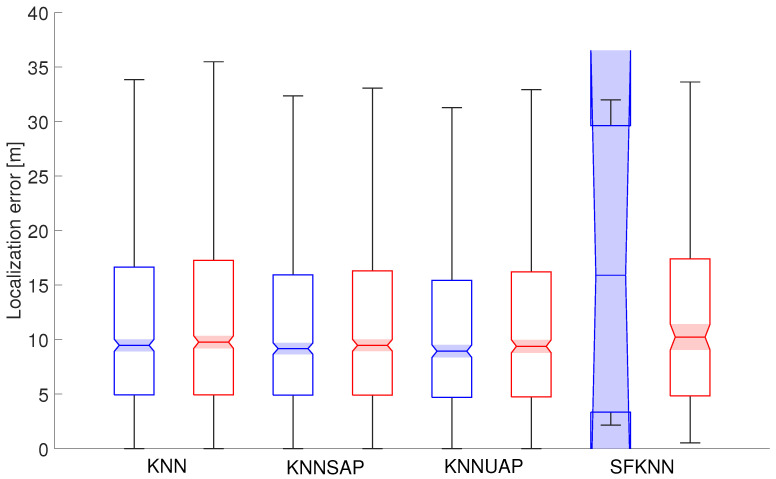
Localization errors achieved in both scenarios with five spoofed APs.

**Figure 6:**
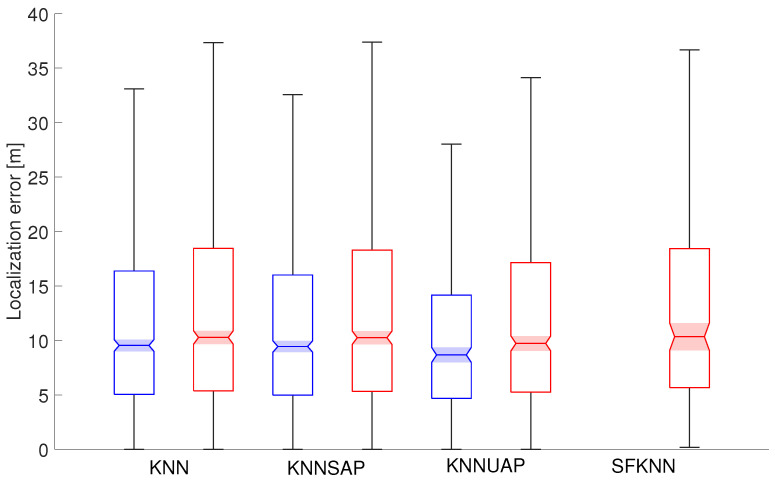
Localization errors achieved in both scenarios with 10 spoofed APs.

Statistical parameters of localization errors achieved for 5 and 10 spoofed APs in both scenarios are shown in Figure 5 and Figure 6, respectively. Boxplots of errors achieved in Scenario 1 are presented in blue color, while errors achieved in Scenario 2 are shown in red.

From Figure 5 it can be seen that the statistical parameters of the localization errors are very similar, which is in line with the mean error values presented in the tables above. The statistical parameters of localization error deviate only for SFKNN in Scenario 1, since the majority of localization requests were filtered out, and therefore, the uncertainty of the achieved median value is extremely large.

On the other hand, when analyzing the data presented in Figure 6 for 10 spoofed APs, it can be seen that the difference between Scenario 1 and Scenario 2 is more significant. SFKNN did filter out all positioning requests in Scenario 1; therefore, there is one boxplot missing in the figure.

Moreover, it should be noted that in Scenario 2 the statistical parameters of localization error seem to be similar for individual algorithms. This is not quite in line with the data presented in Table 5. The major difference is in the number of outliers with large localization errors, which was significantly lower for SFKNN; therefore, the mean error was much lower compared to other algorithms.

### 5.2. Discussion

Based on the achieved results, the KNNSAP solution helped to decrease the localization error compared to the traditional KNN algorithm in Scenario 1 by removing some of the outliers in the localization process. However, the algorithm was not able to drop localization requests because of spoofing. On the other hand, with a more sophisticated spoofing approach in Scenario 2, the localization error increased significantly with a larger number of spoofed APs.

When analyzing the dataset, out of 520 detected APs, only 465 APs were present in the radio map. Therefore, it is clear that 55 new APs were installed in the area between the calibration and localization phases. Moreover, it should also be noted that the average number of detected APs was 18 in the radio map, and 16.5 in the localization requests. Taking into account that more APs were detected during the localization phase, it is clear that the overlap between calibration measurements and localization requests was reduced. This supports the assumption that some APs may have been moved around the campus between the calibration and localization phases.

The function of KNNSAP is affected by the threshold for minimum overlap between APs in localization requests and fingerprints in the radio map. Setting the threshold to a low value could improve the success rate of the localization; however, it may not detect spoofed APs in the localization request. On the other hand, setting the required overlap too high would cause a large number of dropped localization requests in the areas where a small number of APs is detected. The operation of the algorithm is, therefore, affected by the fact that the number of APs detected in the testing dataset is higher compared to the radio map, and at the same time the average number of APs detected in localization requests is smaller than the average number of APs in radio map fingerprints.

On the other hand, KNNUAP achieved localization accuracy comparable with traditional KNN algorithm; however, it was able to remove some localization requests due to spoofing. When the original validation dataset was used, 8% of the requests were denied, which represents a false positive rate. However, it is important to notice that this may be due to the time difference between the collection of training and testing datasets. In Scenario 1, the algorithm was able to detect up to 58% of the spoofed requests, while in the Scenario 2 up to 38% of the spoofed requests, both when 10 spoofed APs were included in requests.

Similar to KNNSAP, the KNNUAP algorithm is affected by the threshold. A low threshold for the difference may affect the success rate of the KNNUAP algorithm, especially taking into account the properties of the used dataset. The overlap between calibration measurements and localization requests was reduced due to the time difference between measurements, the installation of new APs as well as the possible movement of some APs over the localization area. This affected the functionality of all implemented algorithms. However, it should be noted that such a situation could happen in the real implementations of Wi-Fi-based localization systems.

SFKNN was able to detect spoofing in 100% cases in Scenario 1 when the number of spoofed APs was more than six. On the other hand, the detection rate was only between 70% and 78% in scenario 2 when the number of spoofed APs ranged between 3 and 10. It should be noted here, that in Scenario 2, SFKNN achieved the best localization accuracy when more than one spoofed AP was present in the localization request. On the other hand, 12% of the requests were dropped when the original testing dataset, i.e., localization requests without AP spoofing, was used. Therefore, SFKNN had the highest false positive rate. We assume this is due to the parameters of the dataset mentioned above, i.e., the difference in detected APs and decreased the average number of APs in fingerprints. Since some APs have been replaced during the period between the collection of training and testing data, their position estimates from the radio map were not valid anymore. A possible solution for this problem could be the implementation of some threshold for APs without overlap. Such a solution could reduce the false positive rate; however, it may also increase the false negative rate.

## 6. Conclusions and Future Work

In the paper, three solutions were proposed to improve the performance of the KNN algorithm in the case of AP spoofing attacks on the localization system. The performance of these solutions was evaluated using the UJIIndoorLoc dataset, which covers multiple buildings. The validation dataset was altered to simulate the spoofing of beacon signals from APs in two scenarios. The Scenario 1 included signals from a randomly selected AP that was not detected in the original measurements. On the other hand, Scenario 2 spoofing was simulated by copying one or multiple RSS values from the validation dataset that originally belonged to a location in a different building. The SFKNN helped to reduce the localization error in Scenario 2, while in Scenario 1 the detection of spoofing was above 80% when two spoofed APs were present, thus, showing superior parameters to the proposed KNNSAP and KNNUAP algorithms.

In future work, we will focus on improvements to the SFKNN algorithm. It can be assumed that the detection rate of spoofing attacks could be further improved in the Scenario 2. The work will also be focused on the reduction in the false positive rate of the SFKNN algorithm. Testing can also be performed on other datasets; however, there is a lack of datasets covering large areas with multiple buildings. Furthermore, a combination of SFKNN with the filtering of reference points used for position estimation will be investigated. Moreover, the impact of proposed solutions and spoofing of APs on other ML-based methods will be evaluated.

## Figures and Tables

**Table 1:** Root Mean Square Error [m] achieved with different algorithms on UJIIndoorLoc dataset.

Algorithm	RMSE [m]
KNN (baseline)	13.7
CNNLoc [24]	14.86
CHISEL [25]	12.48
SE-Loc [26]	10.56
EA-CNN [27]	10.72
FTLoC [28]	10.45
CNN-LSTM [29]	10.75
VF-CLIP [30]	8.32
DumbLoc [31]	8.5
GNN [32]	9.61

**Table 2:** Overview of spoofing detection algorithms.

Reference	Contribution	Limitation
[39]	Detection of incorrect data in radio map update.	Iterative methods for radio map, not applicable for real-time application.
[40]	Detection of impaired APs in the positioning area.	Detects APs with incorrect RSS, voting algorithm requires updates of radio map.
[15]	Evaluation of Wi-Fi position spoofing and proposed solution for detection.	Use of geolocation based on cellular networks, evaluated on extremely distant positions.
[41]	Detection of spoofed positions using signals from neighbouring nodes	Requires cooperation between individual devices in the area. It does detect spoofed positions, not spoofed signals.
[17]	Detection of spoofed signals based on spatial correlation. Allows to estimate positions of fake APs.	Requires landmarks placed in localization area, which increases the cost of the infrastructure and maintenance.
[18]	Use of CSI measurements for authentication of devices.	Limited access to CSI measurements on off-the-shelf devices.
[19]	Use of CSI measurements and deep learning.	Limited access to CSI measurements on off-the-shelf devices.
[20]	Detection of multiple signals transmitted by a single fake AP.	Requires a large number of RSS samples, thus introducing a significant delay in the localization process.

**Table 3:** Parameters of experimental scenarios.

Parameter	Scenario 1	Scenario 2
Spoofed APs	random	replay of RSS
Number of spoofed APs	1–10	1–10
Localization requests per number of spoofed APs	1111	1111
minimum RSSI of spoofed APs	−70 dBm	−100 dBm
maximum RSSI of spoofed APs	−30 dBm	0 dBm
KNNSAP threshold	5	5
KNNUAP threshold	15	15
*k*-number of RP in position estimation	4	4

**Table 4:** Mean localization errors [m] for different numbers of spoofed APs in Scenario 1.

Algorithm	Number of Spoofed APs									
	**1**	**2**	**3**	**4**	**5**	**6**	**7**	**8**	**9**	**10**
CNNLoc	19.06	22.64	26.92	30.91	33.84	36.27	39.35	41.24	43.70	44.95
KNN	13.79	13.81	13.85	13.88	13.86	13.86	13.86	13.77	13.83	13.75
KNNSAP	11.61	11.63	11.72	11.79	11.84	11.89	11.91	11.91	11.97	12.01
KNNUAP	13.40	13.47	13.34	13.31	13.33	13.37	13.34	13.30	12.07	12.47
SFKNN	13.13	12.10	12.87	14.04	16.48	5.33	NaN	NaN	NaN	NaN

**Table 5:** Mean localization errors [m] for different numbers of spoofed APs in Scenario 2.

Algorithm	Number of Spoofed APs									
	**1**	**2**	**3**	**4**	**5**	**6**	**7**	**8**	**9**	**10**
CNNLoc	16.55	18.95	21.79	24.55	27.54	31.36	32.84	35.98	41.46	40.81
KNN	13.73	13.72	13.77	13.89	14.48	15.24	15.46	17.48	16.91	18.63
KNNSAP	11.65	11.74	11.77	12.08	13.10	14.06	14.36	17.38	17.02	18.42
KNNUAP	13.37	13.34	13.21	13.45	14.04	15.05	15.23	17.30	16.85	18.74
SFKNN	11.99	11.72	12.16	13.02	13.49	14.47	14.50	14.41	14.17	13.73

## Data Availability

Dataset available on request from the authors.
